# Emptiness, one definition, multiple meanings. A qualitative research among young patients in Italian Speaking Switzerland 2022-2025

**DOI:** 10.1192/j.eurpsy.2026.11471

**Published:** 2026-07-31

**Authors:** D. Garino, N. Fascendini, D. Del Maestro

**Affiliations:** Clinica Santa Croce, Orselina, Switzerland

## Abstract

**Introduction:**

Emptiness is a complex and frequently reported experience by individuals with personality disorders, particularly Borderline Personality Disorder, yet its subjective meaning remains multifaceted and poorly understood. While often pathologized, understanding the diverse manifestations of emptiness is crucial for effective therapeutic interventions and person-centered care.

**Objectives:**

This qualitative study aimed to explore and delineate the various subjective interpretations and lived experiences of emptiness among young patients admitted to a psychiatric clinic in Italian-speaking Switzerland. Our goal was to move beyond a singular diagnostic criterion to uncover the richness and heterogeneity of this internal state within a clinical population.

**Methods:**

A qualitative research design was employed, involving in-depth interviews and clinical sessions with a cohort of young patients receiving care at Clinique Santa Croce in Orselina, Switzerland, between 2022 and 2025. Patient selection focused on individuals reporting experiences of emptiness as part of their psychopathology. The interviews were analyzed using thematic analysis to identify recurring patterns and divergent understandings. The exact number of participants is currently being finalized.

**Results:**

Preliminary findings reveal a broad spectrum of meanings associated with emptiness, extending beyond a mere sense of void or absence. Patients described it as a pervasive lack of identity, an emotional numbness, a profound feeling of disconnection from others, a struggle with meaninglessness, or an absence of purpose and direction. These diverse interpretations highlight the highly personal and dynamic nature of emptiness, suggesting that it can manifest as both a painful deficit and, for some, a complex coping or protective state.

**Conclusions:**

This research underscores that emptiness is not a monolithic experience but a heterogeneous phenomenon with profound subjective variations among young psychiatric patients. Recognizing these multiple meanings is vital for clinicians to tailor more nuanced, empathic, and ultimately more effective therapeutic approaches. Further qualitative and quantitative research should explore these distinctions in larger and more diverse populations to refine diagnostic understanding and treatment strategies.

**Disclosure of Interest:**

None Declared

